# Population-Based Prognostic Models for Head and Neck Cancers Using National Cancer Registry Data from Taiwan

**DOI:** 10.1007/s44197-024-00196-7

**Published:** 2024-02-14

**Authors:** Yu-Lun Tsai, Yi-Ting Kang, Han-Ching Chan, Amrita Chattopadhyay, Chun-Ju Chiang, Wen-Chung Lee, Skye Hung-Chun Cheng, Tzu-Pin Lu

**Affiliations:** 1 Institute of Epidemiology and Preventive Medicine, College of Public Health, National Taiwan University, Taipei, Taiwan; 2 Department of Radiation Oncology, Cathay General Hospital, Taipei, Taiwan; 3 Bioinformatics and Biostatistics Core, Center of Genomic and Precision Medicine, National Taiwan University, Taipei, Taiwan; 4 Taiwan Cancer Registry, Taipei, Taiwan; 5 Institute of Health Data Analytics and Statistics, College of Public Health, National Taiwan University, Taipei, Taiwan; 6 Department of Radiation Oncology, Koo Foundation Sun Yat-Sen Cancer Center, Taipei, Taiwan; 7 Taitung Christian Hospital, Taitung, Taiwan

**Keywords:** Prognostic model, Head and neck cancer, Asian, Taiwan Cancer Registry

## Abstract

**Purpose:**

This study aims to raise awareness of the disparities in survival predictions among races in head and neck cancer (HNC) patients by developing and validating population-based prognostic models specifically tailored for Taiwanese and Asian populations.

**Methods:**

A total of 49,137 patients diagnosed with HNCs were included from the Taiwan Cancer Registry (TCR). Six prognostic models, divided into three categories based on surgical status, were developed to predict both overall survival (OS) and cancer-specific survival using the registered demographic and clinicopathological characteristics in the Cox proportional hazards model. The prognostic models underwent internal evaluation through a tenfold cross-validation among the TCR Taiwanese datasets and external validation across three primary racial populations using the Surveillance, Epidemiology, and End Results database. Predictive performance was assessed using discrimination analysis employing Harrell’s c-index and calibration analysis with proportion tests.

**Results:**

The TCR training and testing datasets demonstrated stable and favorable predictive performance, with all Harrell’s c-index values ≥ 0.7 and almost all differences in proportion between the predicted and observed mortality being < 5%. In external validation, Asians exhibited the best performance compared with white and black populations, particularly in predicting OS, with all Harrell’s c-index values > 0.7.

**Conclusions:**

Survival predictive disparities exist among different racial groups in HNCs. We have developed population-based prognostic models for Asians that can enhance clinical practice and treatment plans.

**Supplementary Information:**

The online version contains supplementary material available at 10.1007/s44197-024-00196-7.

## Introduction

Head and neck cancers (HNCs) ranked as the seventh most prevalent form of malignancy worldwide, comprising 4.9% of all cancer cases (931,931 incident cases) and 4.7% of all cancer-related deaths (467,125 deaths) in 2020 [1]. The estimated global burden of disease primarily resulted from years of life lost, accounting for 95.9–98.5% depending on the specific site of cancer. The remaining burden was attributed to years lived with disability, constituting 1.5–4.1% of the total burden [2]. Survival typically takes precedence as the primary concern for individual patients [3]. Prognostic models were developed and validated to predict individualized survival in HNCs [4–6]. These models often relied on data from hospital-based cancer registry systems or medical records in western countries [4–6]. However, survival disparities by race exist in clinical studies, particularly between white and black HNC patients, which have been well documented [7, 8]. The applicability of the current models to other racial populations might be limited.

The Taiwan Cancer Registry (TCR) is a population-based cancer registry that was established by the government of Taiwan in 1979 [9]. The primary purpose is to collect detailed clinical information and demographic characteristics of newly diagnosed cancer patients for surveillance and control efforts [10]. Over time, the TCR has made persistent strides in enhancing the quality and content [9, 10]. The TCR is internationally recognized as one of the highest-quality cancer registries worldwide, providing coverage for nearly 100% of the national population [11]. In academic contexts, the TCR has been utilized to develop prognostic models for several types of cancer, including colon and breast cancers, in Asian populations [12, 13]. Additionally, given its annual registration size of over ten thousand cases of HNCs, the TCR holds substantial potential for developing a reliable prognostic model specifically tailored to HNCs in Asian populations.

This study aimed to develop population-based prognostic models for predicting survival in HNCs. The process involved utilizing demographic, clinical, and pathological information from HNC patients recorded in the TCR between 2013 and 2018 (*N* = 49,137) to construct the models. Furthermore, the models were validated using data from a diverse racial population sourced from the Surveillance, Epidemiology, and End Results (SEER) cancer registry database. This can help shed light on whether race plays a significant role that could potentially influence survival prediction in HNCs. Additionally, it can confirm the effectiveness of the models specifically within Asian populations.

## Materials and methods

**Fig. 1:**
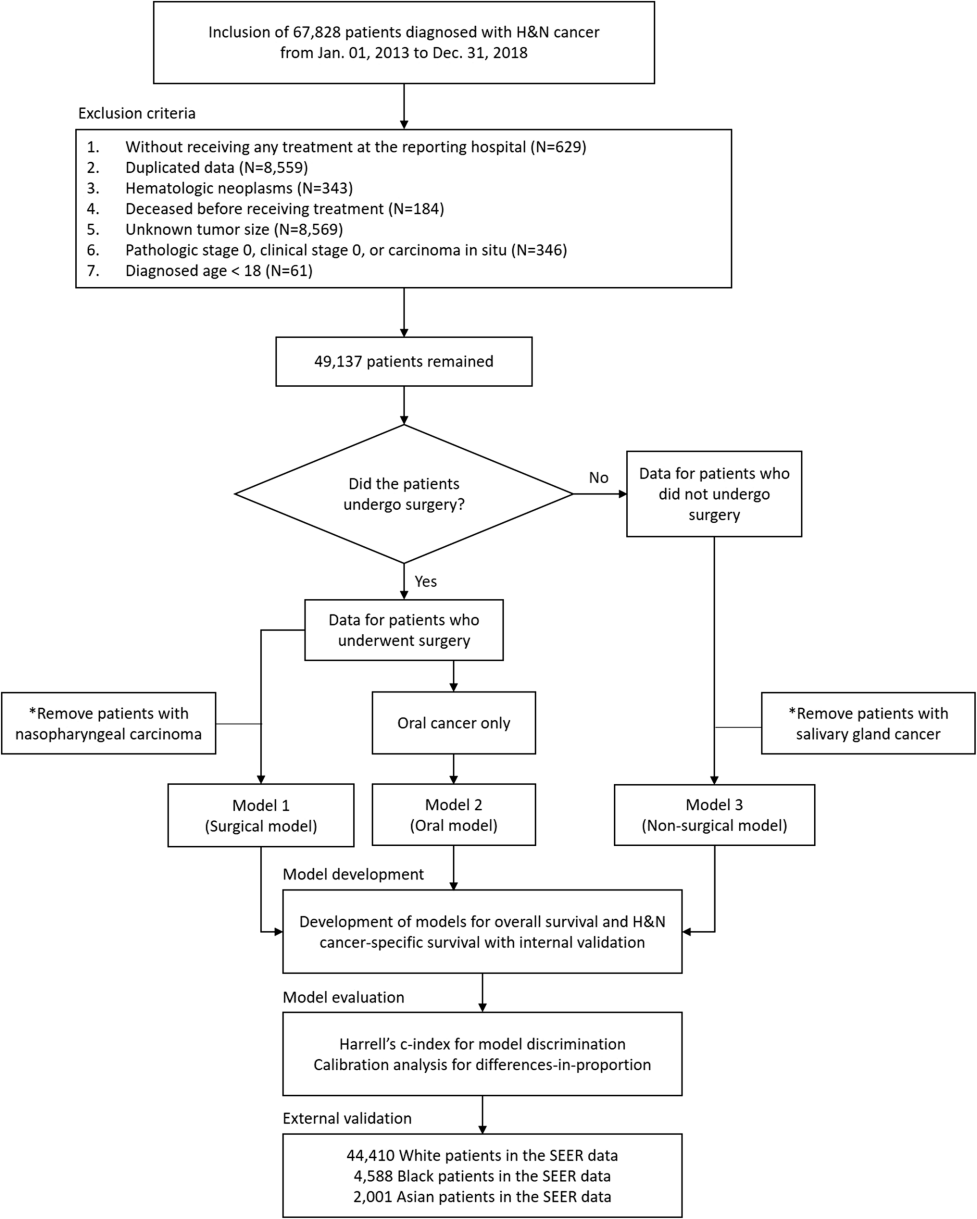
Study process flow diagram

We employed a study methodology that involved patient selection, model development, model evaluation, and external validation. Figure 1 illustrates the entire process flow.

### Patients in Dataset

We collected data on 67,828 patients diagnosed with HNCs between January 01, 2013 and December 31, 2018 from the TCR with follow-up information until December 31, 2020. The definition of HNCs was based on the International Classification of Diseases for Oncology, third edition (ICD-O-3) coding [14]. The tumor site categories included oral cavity (C00: lip, C01–C02: tongue, C03: gum, C04: floor of mouth, C05: palate, C06: other and unspecified parts of mouth), oropharynx (C09–C10), hypopharynx (C12–C13), pharynx and ill-defined sites in lip, oral cavity, and pharynx (C14), salivary glands (C07–C08), nasopharynx (C11), and larynx (C32). We excluded certain patients from the study, including those who did not receive any treatment at the reporting hospital, duplicated data entries, those with hematologic neoplasms, individuals who passed away before receiving treatment, patients with unknown tumor size, those diagnosed with stage 0 or carcinoma in situ, and individuals diagnosed before the age of 18. Finally, a total of 49,137 eligible patients were involved in further analysis. This study has been approved by the institutional review boards of National Taiwan University Hospital (201910027W).

### Variables in Dataset

The variables recorded in the TCR, which have the potential to predict overall survival (OS) and head and neck cancer-specific survival (CSS), were analyzed. These variables include age, sex, body mass index (BMI), diagnosis year, tumor site, tumor size, depth of invasion (DOI), maximum lymph node diameter, the American Joint Committee on Cancer clinical and pathological stage (I, II, III, IV), and treatment modalities including surgery, chemotherapy, and radiotherapy (yes, no). Additionally, behavior factors such as alcohol consumption, tobacco smoking, and betel nut chewing (yes, no) were also considered.

During the final process of external validation, BMI, site-specific features (DOI and maximum lymph node diameter), clinical stage, and behavior factors were excluded from the model since they are not registered in the SEER database. However, race information was included to evaluate prediction differences.

### Model Development

Three types of prognostic models were developed using data from the TCR. Model 1 (surgical model) encompassed all patients who underwent surgery, except those with nasopharyngeal carcinoma. Model 2 (oral model) focused on patients with oral cancer who underwent surgery, representing the majority of surgical patients. Model 3 (non-surgical model) included all non-surgical patients, except those with salivary gland cancer. Each model was constructed using the Cox proportional hazards regression model to estimate the hazards of developing survival events for each variable [15]. Initially, a univariate Cox regression model was employed to identify significant variables, which were further considered as predictors in the multivariate Cox prognostic model. Both OS and head and neck CSS were treated as outcomes in Models 1, 2, and 3, resulting in a total of six prognostic models constructed to comprehensively investigate the model performance of the TCR data for HNCs. All models were built using the 'survival' package in R 4.1.3, a programming language and software environment for statistical computing and graphics, supported by the R Foundation for Statistical Computing [16]. A *P* value of < 0.05 was considered as significant.

### Model Evaluation and Validation

The performance of the prognostic models was first evaluated internally using a tenfold cross-validation, where patients were randomly partitioned into 10 groups, and the averaged performance was calculated [17]. The measurement indicators for model evaluation included model discrimination and calibration [18]. In the discrimination analysis, Harrell’s c-index was used to assess the concordance between the predicted and observed survival status of paired patients [19]. A c-index higher than 0.7 indicates good model performance. As for calibration analysis, the difference in proportion between the predicted and observed mortality of all patients in a given calibration year was statistically tested using the proportion test.

The SEER database served as the external validation dataset in this study, employing the same indicators as used for internal validation, which included discrimination and calibration analyses. The dataset comprised HNC patients diagnosed between 2010 and 2015 in the SEER database [20]. The follow-up period extended until December 31, 2020. The criteria for including the study population were also grounded in the ICD-O-3, and the exclusion criteria for patients were identical to those applied to the TCR dataset mentioned earlier. Race information was available in the SEER database, and three primary populations were examined (Asian, white, and black). Within the Asian population, subpopulations encompassed Chinese, Japanese, Korean, Vietnamese, and Laotian individuals.

## Results

### Patient Characteristics

Table 1 summarizes the demographic and clinicopathological characteristics of the analyzed patients in the TCR and SEER datasets, respectively. Each dataset comprised approximately 50,000 patients, with approximately 60% of the patients having received surgery. Regarding the TCR dataset, the mean age was 56.5, and male patients constituted the majority (88.6%). The number of diagnosed patients showed a slight increase over time, with an annual count of around 8,000. Oral cavity cancer emerged as the most prevalent form of HNCs, encompassing 58.1% of all patients and 78.4% of surgical patients. The tumor size of surgical patients was notably smaller than that of non-surgical patients, with mean sizes of 26.0 mm and 37.7 mm, respectively. Both stages I and IV constituted more than 30% of surgical patients, while stage IV made up the majority, accounting for over 60% of non-surgical patients. Chemotherapy and radiotherapy were administered to only about 40% of surgical patients, whereas approximately 88% of non-surgical patients received these treatments. Three-fourths of the patients had a history of tobacco smoking, whereas only about half reported alcohol consumption and betel nut chewing.

**Table 1:** Demographic and clinicopathological characteristics of the studied datasets

	TCR			SEER		
	Surgical data (*n* = 32,735)	Non-surgical data (*n* = 16,402)	Total (*n* = 49,137)	Surgical data (*n* = 30,305)	Non-surgical data (*n* = 20,694)	Total (*n* = 50,999)
Age (year)	56.5 (11.4)	56.4 (12.2)	56.5 (11.7)	63.1 (13.5)	63.8 (11.8)	63.3 (12.8)
Sex						
Male	29,186 (89.2)	14,328 (87.4)	43,514 (88.6)	20,318 (67.0)	16,274 (78.6)	36,592 (71.8)
Female	3549 (10.8)	2074 (12.6)	5623 (11.4)	9987 (33.0)	4420 (21.4)	14,407 (28.2)
Body mass index	24.7 (4.3)	23.4 (4.2)	24.3 (4.3)	–	–	–
Diagnosis year						
2010	–	–	–	4577 (15.1)	3038 (14.7)	7615 (14.9)
2011	–	–	–	4782 (15.8)	3187 (15.4)	7969 (15.6)
2012	–	–	–	5005 (16.5)	3291 (15.9)	8296 (16.3)
2013	4774 (14.6)	2318 (14.1)	7092 (14.4)	5084 (16.8)	3547 (17.1)	8631 (16.9)
2014	4911 (15.0)	2517 (15.3)	7428 (15.1)	5311 (17.5)	3765 (18.2)	9076 (17.8)
2015	5214 (15.9)	2702 (16.5)	7916 (16.1)	5546 (18.3)	3866 (18.7)	9412 (18.5)
2016	5494 (16.8)	2946 (18.0)	8440 (17.2)	–	–	–
2017	5948 (18.2)	2898 (17.7)	8846 (18.0)	–	–	–
2018	6394 (19.5)	3021 (18.4)	9415 (19.2)	–	–	–
Tumor site						
Oral cavity	25,670 (78.4)	2857 (17.4)	28,527 (58.1)	17,063 (56.3)	8190 (39.6)	25,253 (49.5)
Oropharynx	3102 (9.5)	3879 (23.6)	6981 (14.2)	4765 (15.7)	5570 (26.9)	10,335 (20.3)
Hypopharynx	1446 (4.4)	3290 (20.1)	4736 (9.6)	520 (1.7)	1422 (6.9)	1942 (3.8)
Salivary glands	1361 (4.2)	129 (0.8)	1490 (3.0)	4680 (15.4)	626 (3.0)	5306 (10.4)
Nasopharynx	53 (0.2)	5148 (31.4)	5201 (10.6)	186 (0.6)	1329 (6.4)	1515 (3.0)
Larynx	1103 (3.4)	1099 (6.7)	2202 (4.5)	3091 (10.2)	3557 (17.2)	6648 (13.0)
Tumor size (mm)	26.0 (16.9)	37.7 (18.5)	29.9 (18.3)	24.8 (16.8)	33.7 (17.2)	28.4 (17.5)
Depth of invasion						
1–20 mm	4733 (20.4)	–	–	–	–	–
21–40 mm	3784 (16.3)	–	–	–	–	–
41–60 mm	3102 (13.4)	–	–	–	–	–
61–80 mm	2109 (9.1)	–	–	–	–	–
81–100 mm	1875 (8.1)	–	–	–	–	–
101–120 mm	1284 (5.5)	–	–	–	–	–
121–140 mm	844 (3.6)	–	–	–	–	–
141–160 mm	1148 (5.0)	–	–	–	–	–
≥ 161 mm	3230 (13.9)	–	–	–	–	–
No data available	1080 (4.7)	–	–	–	–	–
Maximum lymph node diameter						
No metastasis	23,991 (75.0)	4080 (28.1)	28,071 (60.3)	–	–	–
1–9 mm	1237 (3.9)	341 (2.3)	1578 (3.4)	–	–	–
10–19 mm	2860 (8.9)	2550 (17.5)	5410 (11.6)	–	–	–
20–29 mm	2077 (6.5)	2664 (18.3)	4741 (10.2)	–	–	–
30–39 mm	916 (2.9)	1766 (12.1)	2682 (5.8)	–	–	–
40–49 mm	358 (1.1)	947 (6.5)	1305 (2.8)	–	–	–
50–59 mm	235 (0.7)	900 (6.2)	1135 (2.4)	–	–	–
≥ 60 mm	305 (1.0)	1293 (8.9)	1598 (3.4)	–	–	–
Pathological stage						
I	11,335 (36.4)	–	–	10,305 (34.4)	–	–
II	6364 (20.5)	–	–	4719 (15.7)	–	–
III	3471 (11.2)	–	–	4748 (15.8)	–	–
IV	9928 (31.9)	–	–	10,217 (34.1)	–	–
Clinical stage						
I	8917 (30.2)	1010 (6.2)	9927 (21.6)	–	–	–
II	6477 (21.9)	2076 (12.7)	8553 (18.6)	–	–	–
III	3472 (11.8)	3015 (18.5)	6487 (14.1)	–	–	–
IV	10,681 (36.1)	10,229 (62.6)	20,910 (45.6)	–	–	–
Chemotherapy						
No	21,794 (66.6)	2021 (12.3)	23,815 (48.5)	22,879 (75.5)	6573 (31.8)	29,452 (57.8)
Yes	10,940 (33.4)	14,381 (87.7)	25,321 (51.5)	7426 (24.5)	14,121 (68.2)	21,547 (42.2)
Radiotherapy						
No	15,226 (57.9)	1515 (11.3)	16,741 (42.2)	14,871 (49.1)	3272 (15.8)	18,143 (35.6)
Yes	11,060 (42.1)	11,854 (88.7)	22,914 (57.8)	15,434 (50.9)	17,422 (84.2)	32,856 (64.4)
Alcohol consumption						
No	17,269 (53.5)	8641 (54.1)	25,910 (53.7)	–	–	–
Yes	15,026 (46.5)	7330 (45.9)	22,356 (46.3)	–	–	–
Tobacco smoking						
No	7216 (22.3)	4494 (27.9)	11,710 (24.1)	–	–	–
Yes	25,183 (77.7)	11,596 (72.1)	36,779 (75.9)	–	–	–
Betel nut chewing						
No	11,545 (35.7)	8363 (52.3)	19,908 (41.2)	–	–	–
Yes	20,765 (64.3)	7618 (47.7)	28,383 (58.8)	–	–	–
Race						
White	–	–	–	26,909 (88.8)	17,501 (84.6)	44,410 (87.1)
Black	–	–	–	2234 (7.4)	2354 (11.4)	4588 (9.0)
Asian	–	–	–	1162 (3.8)	839 (4.1)	2001 (3.9)

*TCR* Taiwan Cancer Registry; *SEER* Surveillance, Epidemiology, and End Results database

Depth of invasion is measured from the level of the basement membrane of the closest adjacent normal mucosa. Maximum lymph node diameter corresponds to the diameter of the largest metastatic lymph node in the neck

All data are presented as *n* (%) except for age, body mass index, and tumor size, which are reported as mean values (standard error)

In the context of the SEER dataset, the composition exhibited similarities with the TCR dataset. The mean age was 63.3, with male patients constituting the majority (71.8%). Diagnosed patient counts averaged around 8000 annually, showing a slight upward trend over time. Oral cavity cancer stood out as the most prevalent HNC type, representing 49.5% of all patients and 56.3% of surgical patients. Notably different mean tumor sizes were observed between surgical (24.8 mm) and non-surgical patients (33.7 mm). Pathological stage information revealed that both stages I and IV accounted for more than 30% of surgical patients. The administration rates of chemotherapy and radiotherapy were significantly higher among non-surgical patients. In terms of race information, the dataset was predominantly composed of patients identifying as white (87.1%).

### Prognostic Models

Six prognostic models were developed to predict both OS and CSS for three categories of surgical status: surgical patients (Model 1), surgical oral cancer patients (Model 2), and non-surgical patients (Model 3). Table 2 provides an overview of the significant variables identified in the univariate Cox regression model, along with their hazard ratios (HRs) and associated statistical significances estimated in the multivariate Cox prognostic model for Model 1 with respect to OS. The summaries of the remaining five models are presented in Supplementary Tables S1–S5. Generally, older age, male sex, lower BMI, and earlier diagnosis year were associated with increased hazards of mortality. Oropharyngeal cancer among surgical patients, palate cancer among patients with surgical oral cavity cancer, and oral cavity cancer among non-surgical patients were linked to poorer survival outcomes. Larger tumor size, greater maximum lymph node diameter, and higher clinical and pathological stages were identified as adverse prognostic factors. DOI was significant only in Model 2 pertaining to surgical oral cavity cancer. Chemotherapy had a slightly negative impact on survival in Model 1 for both OS and CSS, as well as in Model 3 for OS. In contrast, radiotherapy exhibited positive effects in all models. Alcohol consumption demonstrated detrimental effects across all models, while tobacco smoking showed a contrasting effect in Model 1.

**Table 2:** Results of Cox regression Model 1 (surgical model) for overall survival

	Univariate		Multivariate	
	HR (95% CI)	*P* value	HR (95% CI)	*P* value
Age (per year)	1.02 (1.02–1.02)	< 0.001	1.02 (1.02–1.02)	< 0.001
Sex				
Male	–		–	
Female	0.72 (0.67–0.77)	< 0.001	0.74 (0.68–0.81)	< 0.001
Body mass index (per score)	0.93 (0.93–0.94)	< 0.001	0.95 (0.95–0.96)	< 0.001
Diagnosis year (per year)	0.97 (0.95–0.98)	< 0.001	0.97 (0.95–0.98)	< 0.001
Tumor site				
Oral cavity	–		–	
Oropharynx	1.47 (1.38–1.56)	< 0.001	1.24 (1.16–1.33)	< 0.001
Hypopharynx	1.9 (1.76–2.04)	< 0.001	0.9 (0.82–0.98)	0.020
Salivary glands	0.62 (0.55–0.7)	< 0.001	0.72 (0.62–0.83)	< 0.001
Larynx	1.01 (0.91–1.13)	0.794	0.92 (0.81–1.04)	0.162
Tumor size (per mm)	1.02 (1.02–1.02)	< 0.001	1.01 (1.01–1.01)	< 0.001
Maximum lymph node diameter				
No metastasis	–		–	
1–9 mm	2.15 (1.97–2.35)	< 0.001	1.89 (1.7–2.11)	< 0.001
10–19 mm	2.55 (2.41–2.7)	< 0.001	1.94 (1.8–2.1)	< 0.001
20–29 mm	2.74 (2.57–2.92)	< 0.001	2.02 (1.86–2.2)	< 0.001
30–39 mm	3.16 (2.89–3.45)	< 0.001	2.17 (1.95–2.43)	< 0.001
40–49 mm	2.96 (2.57–3.4)	< 0.001	1.89 (1.6–2.23)	< 0.001
50–59 mm	3.25 (2.75–3.85)	< 0.001	2.18 (1.79–2.66)	< 0.001
≥ 60 mm	4.16 (3.62–4.78)	< 0.001	2.76 (2.35–3.24)	< 0.001
Pathological stage				
I	–		–	
II	1.35 (1.26–1.43)	< 0.001	1.09 (1.01–1.17)	0.020
III	1.79 (1.67–1.93)	< 0.001	0.97 (0.88–1.07)	0.561
IV	3.34 (3.18–3.51)	< 0.001	1.52 (1.39–1.66)	< 0.001
Chemotherapy				
No	–		–	
Yes	2.04 (1.96–2.12)	< 0.001	1.1 (1.03–1.17)	0.003
Radiotherapy				
No	–		–	
Yes	1.83 (1.76–1.91)	< 0.001	0.81 (0.76–0.87)	< 0.001
Alcohol consumption				
No	–		–	
Yes	1.34 (1.29–1.39)	< 0.001	1.22 (1.17–1.28)	< 0.001
Tobacco smoking				
No	–		–	
Yes	1.13 (1.08–1.19)	< 0.001	0.89 (0.84–0.95)	< 0.001

*HR* Hazard ratio; *CI* Confidence interval

### Performance of the Models

The predictive performance of the models was assessed through internal and external validations, which included both discrimination and calibration analyses. Table 3 presents the results of discrimination analysis, encompassing the TCR training and testing datasets in internal validation, and the three primary populations from the SEER dataset in external validation. Generally, Harrell’s *c*-index exhibited stability in internal validation, with values exceeding 0.7 for both OS and CSS in both the training and testing datasets in all models. For external validation, the *c*-index generally ranged between 0.6 and 0.7. However, the values exceeded 0.7 for the Asian population when predicting OS in all models and CSS in Model 3, suggesting that the models are particularly effective for the Asian population.

**Table 3:** Harrell’s c-index values in discrimination analysis for overall survival and cancer-specific survival in TCR datasets (training and testing datasets) and SEER datasets (Asian, White, and Black populations)

Model	End point	Internal validation (TCR)		External validation (SEER)			
		Training	Testing	Asian	White	Black	Total
Model 1	OS	0.70	0.70	0.73	0.65	0.66	0.65
	CSS	0.76	0.77	0.68	0.63	0.63	0.63
Model 2	OS	0.71	0.71	0.74	0.70	0.68	0.70
	CSS	0.78	0.79	0.66	0.62	0.61	0.62
Model 3	OS	0.74	0.74	0.74	0.66	0.68	0.66
	CSS	0.78	0.79	0.72	0.60	0.63	0.61

*TCR* Taiwan Cancer Registry; *SEER* Surveillance, Epidemiology, and End Results database; *OS* Overall survival; *CSS* Cancer-specific survival

Model 1: surgical model; Model 2: oral model; Model 3: non-surgical model

Regarding calibration analysis, the objective is to assess whether the predicted number of death events significantly differs from the observed number of death events. Table 4 presents the results for Model 1 concerning OS, while the results for Models 2 and 3 for OS, and all three models for CSS are summarized in Supplementary Tables S6–S10. Across different calibration years, the majority of differences in proportion between predicted and observed deaths were less than 5% in both the TCR training and testing datasets, accompanied by insignificant *P* values for all models (Table 4 and Supplementary Tables S6–S10). This suggests that the models perform exceptionally well in predicting both overall and cancer-specific mortalities in the Taiwanese population. When considering the three primary populations in the SEER dataset, Model 1 demonstrated outstanding predictive capability for OS, with the majority of differences in proportion being less than 5%, especially for the Asian and black populations, which were associated with insignificant *P* values (Table 4). Model 2 exhibited satisfactory predictive capability with differences in proportion below 10% for both OS and CSS (Supplementary Tables S6 and S9). In contrast, Models 3 performed relatively poorly, especially for the white population, where some differences in proportion exceeded 20% (Supplementary Tables S7 and S10).

**Table 4:** Results of calibration analysis for Model 1 (surgical model) for overall mortality in different populations

Calibration year	No. of cases	Observed	Predicted	Difference (%)	*P* value
Training					
1	21,298	2314	2347	0.155	0.604
2	18,984	4561	4632	0.374	0.393
3	16,737	5731	5804	0.436	0.398
4	11,946	5156	5212	0.469	0.458
5	8180	4207	4244	0.452	0.559
Testing					
1	2366	252	261	0.380	0.672
2	2114	538	516	− 1.041	0.435
3	1828	658	646	− 0.656	0.703
4	1302	605	588	− 1.306	0.511
5	886	473	471	− 0.226	0.956
Asian					
1	1086	112	127	1.381	0.282
2	1075	184	247	5.860	< 0.001
3	1061	243	303	5.655	0.003
4	1031	288	339	4.947	0.014
5	870	316	325	1.034	0.630
White					
1	26,325	2888	3361	1.797	< 0.001
2	26,197	5440	6564	4.291	< 0.001
3	26,078	7014	8104	4.180	< 0.001
4	25,630	8252	9105	3.328	< 0.001
5	22,441	9274	8919	− 1.582	< 0.001
Black					
1	2182	326	292	− 1.558	0.148
2	2174	562	568	0.276	0.834
3	2166	731	699	− 1.477	0.303
4	2137	832	785	− 2.199	0.141
5	1905	913	781	− 6.929	< 0.001

Differences are calculated as ((predicted deaths − observed deaths)/number of cases) × 100

## Discussion

This study introduced three types of prognostic models designed for three groups of HNC patients: surgical patients (Model 1), surgical oral cancer patients (Model 2), and non-surgical patients (Model 3), with the aim of predicting both OS and CSS. The development of these models was grounded in data from the national cancer registry of Taiwan, encompassing 49,137 patients diagnosed with HNCs between 2013 and 2018. The discrimination and calibration analyses during internal validation consistently exhibited remarkable performance in predicting HNC survival and mortality across all models and settings within the Taiwanese population. When testing these models on the SEER database for different populations during external validation, we observed that race significantly impacts survival prediction in HNCs. The models were most effective for the Asian population, particularly in predicting OS for surgical patients. These results affirm the efficacy of the presented models in Asian populations, underscoring the importance of developing tailored models for specific demographic groups.

The variables included in the models consisted of only 15 items from the cancer registry database, excluding information that is difficult to derive. This could enhance the practicality and applicability of the research findings, similar to how PREDICT, a valuable prognostic algorithm for predicting OS and CSS in early-stage breast cancer in Britain, evolved into a useful online predictive tool [21, 22]. The American Joint Committee on Cancer (AJCC) has acknowledged the demand for more personalized probabilistic predictions and has established criteria for the inclusion and exclusion of risk models in individualized prognosis as part of the practice of precision medicine [23]. The AJCC acceptance criteria have been applied to review early published prognostic nomograms in HNCs [24]. The prognostic models we introduced adhere to the AJCC criteria to enhance the integrity of research results, as well as their practical applicability.

An important point to clarify is that the effects of the variables presented in the prognostic models, as indicated by the HR in Table 2 and Supplementary Tables S1–S5, may not definitively represent the unbiased, pure effects of these variables. These effects were accurately estimated using the exponential of the coefficients in the Cox regression model. However, the causal diagram becomes significantly complex in clinical scenarios when multiple levels and domains of variables are included. Undiscovered associations or interactions among these variables may lead to overestimation or underestimation of the pure effects. Moreover, although we selected the 15 most relevant variables from the TCR dataset based on our knowledge, there is a probability that other factors may be underestimated or even unaccounted for. In this study, the overall predictive capability of the models holds more significance than the individual effect of each variable. Further assessment of these effects may be conducted in dedicated studies that control for confounding factors.

Besides the available information from the registry data we employed, other prognostic models for HNCs have been developed by incorporating predictive factors from various fields. These factors include autophagy-related genes [25], cancer stem cell markers [26], immune signatures [27], parameters from positron emission tomography/computed tomography (PET/CT) [28], and radiomic features from magnetic resonance (MR) or CT scans [29–31]. Greater efforts are needed to obtain this information since it is typically not collected as part of routine procedures, but it has the potential to enhance the predictive capabilities of the models. Concerning specific types of cancer, there has been an increase in human papillomavirus (HPV)-associated oropharyngeal cancer, primarily induced by HPV type 16, among younger individuals [32]. Patients with HPV-associated oropharyngeal cancer tend to exhibit increased sensitivity to chemoradiotherapy, resulting in improved survival rates [32]. To predict outcomes for oral cavity cancer, machine learning algorithms have been employed in the development of prediction models [33–35].

This study possesses several strengths. First, a relatively large sample size is involved, encompassing 49,137 HNC patients from the TCR, enhancing the precision of the model predictions. Second, the models have been confirmed to be effective within the Taiwanese and Asian populations, highlighting the significance of personalized survival predictions that take race into account in HNCs. Third, the models utilize only 15 commonly registered items as predictive variables, enhancing their clinical applicability and practicality.

This study also has some limitations. First, the performance of the models still has room for improvement, especially in predicting CSS, as some of the Harrell’s c-index values in discrimination analysis are below 0.7, and differences in proportion in calibration analysis exceed 10%. Second, despite the SEER database being one of the most comprehensive databases, covering approximately 50% of the US population, the SEER dataset used for external validation is predominantly composed of white patients. To enhance the robustness of our findings, the next step could involve exploring other databases to confirm the predictive efficacy in Asian populations. Third, distinguishing whether the racial disparities observed in model predictions in this study genuinely result from racial or ethnic backgrounds is challenging and necessitates cultural and social information to ascertain the underlying factors.

## Conclusions

In summary, we have developed and validated population-based prognostic models for predicting HNC survival using the TCR data with a large sample size from Taiwan. In internal validation, the models exhibited favorable and stable performance among the Taiwanese population. In external validation, the models consistently demonstrated satisfactory performance in predicting OS in Asians. Our results emphasize the importance of tailored survival models for specific demographic groups, and provide practically applicable models for Asian HNC patients.

### Supplementary Information

Below is the link to the electronic supplementary material.

Supplementary file1 (DOCX 44 kb)

## Data Availability

The datasets used and/or analyzed during the current study are available from the corresponding author on reasonable request.

## Abbreviations

HNC: Head and neck cancer
TCR: Taiwan Cancer Registry
SEER: Surveillance, Epidemiology, and End Results
OS: Overall survival
CSS: Cancer-specific survival
BMI: Body mass index
DOI: Depth of invasion
HR: Hazard ratio
AJCC: American Joint Committee on Cancer
PET: Positron emission tomography
CT: Computed tomography
MR: Magnetic resonance
HPV: Human papillomavirus
