# Supplementary material for: Population-Based Prognostic Models for Head and Neck Cancers Using National Cancer Registry Data from Taiwan
Source: J Epidemiol Glob Health. 2024 Feb 14;14(2):433–43. doi: 10.1007/s44197-024-00196-7 (PMC11176144; doi:10.1007/s44197-024-00196-7)
Supplement: Supplementary file 1 — Supplementary file1 (DOCX 44 kb) [file 44197_2024_196_MOESM1_ESM.docx]

**Supplementary Tables**

Table S1. Results of Cox regression Model 2 (oral model) for overall survival.

Table S2. Results of Cox regression Model 3 (non-surgical model) for overall survival.

Table S3. Results of Cox regression Model 1 (surgical model) for cancer-specific survival.

Table S4. Results of Cox regression Model 2 (oral model) for cancer-specific survival.

Table S5. Results of Cox regression Model 3 (non-surgical model) for cancer-specific survival.

Table S6. Results of calibration analysis for Model 2 (oral model) for overall mortality in different populations.

Table S7. Results of calibration analysis for Model 3 (non-surgical model) for overall mortality in different populations.

Table S8. Results of calibration analysis for Model 1 (surgical model) for cancer-specific mortality in different populations.

Table S9. Results of calibration analysis for Model 2 (oral model) for cancer-specific mortality in different populations.

Table S10. Results of calibration analysis for Model 3 (non-surgical model) for cancer-specific mortality in different populations.

Table S1. Results of Cox regression Model 2 (oral model) for overall survival.

|  | Univariate | |  | Multivariate | |
| --- | --- | --- | --- | --- | --- |
|  | HR (95% CI) | *P* value |  | HR (95% CI) | *P* value |
| Age (per year) | 1.02 (1.02-1.02) | <.001 |  | 1.02 (1.02 - 1.02) | <.001 |
| Sex |  |  |  |  |  |
| Male | – |  |  | – |  |
| Female | 0.87 (0.81-0.94) | 0.001 |  | 0.84 (0.77 - 0.93) | <.001 |
| Body mass index (per score) | 0.94 (0.93-0.94) | <.001 |  | 0.96 (0.95 - 0.97) | <.001 |
| Diagnosis year (per year) | 0.97 (0.96-0.98) | <.001 |  | 0.97 (0.95 - 0.98) | <.001 |
| Tumor site |  |  |  |  |  |
| Lip | – |  |  | – |  |
| Tongue | 1.1 (1-1.21) | 0.057 |  | 0.94 (0.84 - 1.06) | 0.331 |
| Gum | 1.38 (1.25-1.53) | <.001 |  | 0.98 (0.86 - 1.11) | 0.735 |
| Floor of mouth | 1.27 (1.1-1.45) | 0.001 |  | 0.96 (0.81 - 1.13) | 0.590 |
| Palate | 1.45 (1.25-1.69) | <.001 |  | 1.28 (1.06 - 1.55) | 0.010 |
| Other and unspecified parts | 0.99 (0.9-1.09) | 0.852 |  | 0.87 (0.77 - 0.97) | 0.016 |
| Tumor size (per mm) | 1.02 (1.02-1.02) | <.001 |  | 1.01 (1.01 - 1.01) | <.001 |
| Depth of invasion |  |  |  |  |  |
| 1-20 mm | – |  |  | – |  |
| 21-40 mm | 1.37 (1.25-1.51) | <.001 |  | 1.23 (1.1 - 1.37) | <.001 |
| 41-60 mm | 1.7 (1.54-1.87) | <.001 |  | 1.41 (1.26 - 1.58) | <.001 |
| 61-80 mm | 2.02 (1.82-2.24) | <.001 |  | 1.57 (1.39 - 1.78) | <.001 |
| 81-100 mm | 2.27 (2.04-2.52) | <.001 |  | 1.55 (1.36 - 1.76) | <.001 |
| 101-120 mm | 2.68 (2.4-3) | <.001 |  | 1.75 (1.52 - 2.01) | <.001 |
| 121-140 mm | 2.87 (2.53-3.26) | <.001 |  | 1.8 (1.54 - 2.09) | <.001 |
| 141-160 mm | 3.47 (3.11-3.87) | <.001 |  | 1.88 (1.64 - 2.15) | <.001 |
| ≥ 161 mm | 4.1 (3.77-4.47) | <.001 |  | 1.98 (1.75 - 2.24) | <.001 |
| No data available | 2.78 (2.47-3.13) | <.001 |  | 1.48 (1.27 - 1.74) | <.001 |
| Maximum lymph node diameter |  |  |  |  |  |
| No metastasis | – |  |  | – |  |
| 1-9 mm | 2.28 (2.07-2.5) | <.001 |  | 2.13 (1.88 - 2.42) | <.001 |
| 10-19 mm | 2.73 (2.56-2.91) | <.001 |  | 2.26 (2.06 - 2.47) | <.001 |
| 20-29 mm | 3.12 (2.9-3.37) | <.001 |  | 2.38 (2.15 - 2.64) | <.001 |
| 30-39 mm | 4 (3.58-4.48) | <.001 |  | 3.19 (2.78 - 3.67) | <.001 |
| 40-49 mm | 3.97 (3.27-4.81) | <.001 |  | 2.7 (2.14 - 3.41) | <.001 |
| 50-59 mm | 4.1 (3.25-5.18) | <.001 |  | 2.87 (2.15 - 3.83) | <.001 |
| ≥ 60 mm | 4.37 (3.64-5.25) | <.001 |  | 3.34 (2.7 - 4.14) | <.001 |
| Pathological stage |  |  |  |  |  |
| I | – |  |  | – |  |
| II | 1.38 (1.29-1.49) | <.001 |  | 0.94 (0.86 - 1.03) | 0.180 |
| III | 1.87 (1.72-2.03) | <.001 |  | 0.74 (0.65 - 0.85) | <.001 |
| IV | 3.52 (3.33-3.72) | <.001 |  | 1.19 (1.06 - 1.34) | 0.004 |
| Radiotherapy |  |  |  |  |  |
| No | – |  |  | – |  |
| Yes | 2.03 (1.94-2.13) | <.001 |  | 0.84 (0.77 - 0.9) | <.001 |
| Alcohol consumption |  |  |  |  |  |
| No | – |  |  | – |  |
| Yes | 1.25 (1.2-1.3) | <.001 |  | 1.17 (1.11 - 1.23) | <.001 |
| *Abbreviations: HR* Hazard ratio; *CI* Confidence interval. | | | | | |

Table S2. Results of Cox regression Model 3 (non-surgical model) for overall survival.

|  | Univariate | |  | Multivariate | |
| --- | --- | --- | --- | --- | --- |
|  | HR (95% CI) | *P* value |  | HR (95% CI) | *P* value |
| Age (per year) | 1.03 (1.03-1.03) | <.001 |  | 1.02 (1.02 - 1.02) | <.001 |
| Sex |  |  |  |  |  |
| Male | – |  |  | – |  |
| Female | 0.72 (0.67 - 0.77) | <.001 |  | 0.78 (0.7 - 0.86) | <.001 |
| Body mass index (per score) | 0.91 (0.9 - 0.91) | <.001 |  | 0.94 (0.93 - 0.95) | <.001 |
| Diagnosis year (per year) | 0.97 (0.95 - 0.98) | <.001 |  | 0.97 (0.95 - 0.98) | <.001 |
| Tumor site |  |  |  |  |  |
| Oral cavity | – |  |  | – |  |
| Oropharynx | 0.51 (0.48 - 0.54) | <.001 |  | 0.67 (0.62 - 0.73) | <.001 |
| Hypopharynx | 0.61 (0.58 - 0.65) | <.001 |  | 0.77 (0.71 - 0.83) | <.001 |
| Nasopharynx | 0.16 (0.15 - 0.17) | <.001 |  | 0.35 (0.32 - 0.39) | <.001 |
| Larynx | 0.33 (0.3 - 0.36) | <.001 |  | 0.54 (0.48 - 0.62) | <.001 |
| Tumor size (per mm) | 1.02 (1.02 - 1.02) | <.001 |  | 1.01 (1.01 - 1.01) | <.001 |
| Maximum lymph node diameter |  |  |  |  |  |
| No metastasis | – |  |  | – |  |
| 1-9 mm | 0.79 (0.67 - 0.93) | 0.005 |  | 0.88 (0.73 - 1.06) | 0.005 |
| 10-19 mm | 1.08 (1.01 - 1.15) | 0.028 |  | 0.88 (0.81 - 0.95) | 0.028 |
| 20-29 mm | 1.04 (0.97 - 1.11) | 0.270 |  | 1.00 (0.92 - 1.09) | 0.270 |
| 30-39 mm | 0.95 (0.88 - 1.03) | 0.228 |  | 1.03 (0.93 - 1.13) | 0.228 |
| 40-49 mm | 1.07 (0.97 - 1.18) | 0.170 |  | 1.26 (1.12 - 1.41) | 0.170 |
| 50-59 mm | 1.14 (1.04 - 1.26) | 0.008 |  | 1.21 (1.08 - 1.36) | 0.008 |
| ≥ 60 mm | 1.53 (1.42 - 1.66) | <.001 |  | 1.55 (1.4 - 1.71) | <.001 |
| Clinical stage |  |  |  |  |  |
| I | – |  |  | – |  |
| II | 1.01 (0.88 - 1.16) | 0.855 |  | 1.18 (1.01 - 1.39) | 0.855 |
| III | 1.23 (1.08 - 1.4) | 0.002 |  | 1.52 (1.29 - 1.78) | 0.002 |
| IV | 3.23 (2.88 - 3.63) | <.001 |  | 2 (1.71 - 2.34) | <.001 |
| Chemotherapy |  |  |  |  |  |
| No | – |  |  | – |  |
| Yes | 0.84 (0.79 - 0.89) | <.001 |  | 0.66 (0.6 - 0.72) | <.001 |
| Radiotherapy |  |  |  |  |  |
| No | – |  |  | – |  |
| Yes | 0.3 (0.29 - 0.32) | <.001 |  | 0.5 (0.46 - 0.53) | <.001 |
| Alcohol consumption |  |  |  |  |  |
| No | – |  |  | – |  |
| Yes | 1.71 (1.64 - 1.78) | <.001 |  | 1.25 (1.18 - 1.32) | <.001 |
| *Abbreviations: HR* Hazard ratio; *CI* Confidence interval. | | | | | |

Table S3. Results of Cox regression Model 1 (surgical model) for cancer-specific survival.

|  | Univariate | |  | Multivariate | |
| --- | --- | --- | --- | --- | --- |
|  | HR (95% CI) | *P* value |  | HR (95% CI) | *P* value |
| Sex |  |  |  |  |  |
| Male | – |  |  | – |  |
| Female | 0.68 (0.61 - 0.77) | <.001 |  | 0.76 (0.66 - 0.88) | <.001 |
| Body mass index (per score) | 0.94 (0.93 - 0.94) | <.001 |  | 0.96 (0.95 - 0.97) | <.001 |
| Diagnosis year (per year) | 0.63 (0.62 - 0.65) | <.001 |  | 0.7 (0.68 - 0.72) | <.001 |
| Tumor site |  |  |  |  |  |
| Oral cavity | – |  |  | – |  |
| Oropharynx | 1.44 (1.31 - 1.59) | <.001 |  | 1.16 (1.04 - 1.29) | 0.009 |
| Hypopharynx | 1.93 (1.71 - 2.18) | <.001 |  | 0.78 (0.68 - 0.89) | <.001 |
| Salivary glands | 0.63 (0.52 - 0.77) | <.001 |  | 0.73 (0.58 - 0.91) | 0.004 |
| Larynx | 0.81 (0.66 - 0.98) | 0.030 |  | 0.85 (0.69 - 1.05) | 0.139 |
| Tumor size (per mm) | 1.03 (1.03 - 1.03) | <.001 |  | 1.01 (1.01 - 1.02) | <.001 |
| Maximum lymph node diameter |  |  |  |  |  |
| No metastasis | – |  |  | – |  |
| 1-9 mm | 2.3 (1.98 - 2.67) | <.001 |  | 1.97 (1.67 - 2.32) | <.001 |
| 10-19 mm | 3.62 (3.31 - 3.95) | <.001 |  | 2.29 (2.05 - 2.57) | <.001 |
| 20-29 mm | 4.07 (3.7 - 4.49) | <.001 |  | 2.58 (2.28 - 2.92) | <.001 |
| 30-39 mm | 4.45 (3.89 - 5.09) | <.001 |  | 2.46 (2.1 - 2.88) | <.001 |
| 40-49 mm | 4.07 (3.29 - 5.04) | <.001 |  | 1.99 (1.56 - 2.53) | <.001 |
| 50-59 mm | 5.06 (3.97 - 6.46) | <.001 |  | 2.86 (2.2 - 3.71) | <.001 |
| ≥ 60 mm | 5.84 (4.75 - 7.18) | <.001 |  | 2.93 (2.33 - 3.68) | <.001 |
| Pathological stage |  |  |  |  |  |
| I | – |  |  | – |  |
| II | 1.51 (1.35 - 1.7) | <.001 |  | 1.26 (1.11 - 1.42) | <.001 |
| III | 2.21 (1.94 - 2.5) | <.001 |  | 1.14 (0.97 - 1.34) | 0.104 |
| IV | 5.01 (4.59 - 5.48) | <.001 |  | 2.19 (1.9 - 2.52) | <.001 |
| Chemotherapy |  |  |  |  |  |
| No | – |  |  | – |  |
| Yes | 2.72 (2.56 - 2.9) | <.001 |  | 1.17 (1.06 - 1.29) | 0.001 |
| Radiotherapy |  |  |  |  |  |
| No | – |  |  | – |  |
| Yes | 2.36 (2.21 - 2.52) | <.001 |  | 0.71 (0.64 - 0.79) | <.001 |
| Alcohol consumption |  |  |  |  |  |
| No | – |  |  | – |  |
| Yes | 1.29 (1.21 - 1.37) | <.001 |  | 1.14 (1.06 - 1.23) | <.001 |
| Tobacco smoking |  |  |  |  |  |
| No | – |  |  | – |  |
| Yes | 1.14 (1.05 - 1.23) | 0.001 |  | 0.85 (0.77 - 0.93) | 0.001 |
| *Abbreviations: HR* Hazard ratio; *CI* Confidence interval. | | | | | |

Table S4. Results of Cox regression Model 2 (oral model) for cancer-specific survival.

|  | Univariate | |  | Multivariate | |
| --- | --- | --- | --- | --- | --- |
|  | HR (95% CI) | *P* value |  | HR (95% CI) | *P* value |
| Body mass index (per score) | 0.94 (0.94 - 0.95) | <.001 |  | 0.97 (0.96 - 0.98) | <.001 |
| Diagnosis year (per year) | 0.64 (0.62 - 0.65) | <.001 |  | 0.69 (0.67 - 0.72) | <.001 |
| Tumor site |  |  |  |  |  |
| Lip | – |  |  | – |  |
| Tongue | 1.44 (1.2 - 1.72) | <.001 |  | 0.94 (0.77 - 1.15) | 0.533 |
| Gum | 1.8 (1.49 - 2.17) | <.001 |  | 1.1 (0.89 - 1.36) | 0.389 |
| Floor of mouth | 1.6 (1.25 - 2.05) | <.001 |  | 0.96 (0.73 - 1.27) | 0.776 |
| Palate | 2.11 (1.63 - 2.72) | <.001 |  | 1.65 (1.24 - 2.21) | 0.001 |
| Other and unspecified parts | 1.37 (1.14 - 1.64) | <.001 |  | 0.97 (0.8 - 1.19) | 0.787 |
| Tumor size (per mm) | 1.03 (1.03 - 1.03) | <.001 |  | 1.01 (1.01 - 1.01) | <.001 |
| Depth of invasion |  |  |  |  |  |
| 1-20 mm | – |  |  | – |  |
| 21-40 mm | 1.73 (1.43 - 2.1) | <.001 |  | 1.52 (1.24 - 1.87) | <.001 |
| 41-60 mm | 2.59 (2.15 - 3.12) | <.001 |  | 1.91 (1.56 - 2.35) | <.001 |
| 61-80 mm | 3.12 (2.57 - 3.8) | <.001 |  | 2.17 (1.75 - 2.69) | <.001 |
| 81-100 mm | 3.71 (3.05 - 4.51) | <.001 |  | 2.14 (1.72 - 2.66) | <.001 |
| 101-120 mm | 4.43 (3.61 - 5.45) | <.001 |  | 2.49 (1.97 - 3.14) | <.001 |
| 121-140 mm | 5.18 (4.15 - 6.47) | <.001 |  | 2.41 (1.87 - 3.1) | <.001 |
| 141-160 mm | 5.93 (4.86 - 7.24) | <.001 |  | 2.53 (2.01 - 3.19) | <.001 |
| ≥ 161 mm | 7.46 (6.32 - 8.8) | <.001 |  | 2.67 (2.16 - 3.3) | <.001 |
| No data available | 5.44 (4.43 - 6.67) | <.001 |  | 2.04 (1.59 - 2.63) | <.001 |
| Maximum lymph node diameter |  |  |  |  |  |
| No metastasis | – |  |  | – |  |
| 1-9 mm | 2.41 (2.05 - 2.85) | <.001 |  | 2.34 (1.93 - 2.83) | <.001 |
| 10-19 mm | 4.12 (3.74 - 4.55) | <.001 |  | 2.91 (2.55 - 3.33) | <.001 |
| 20-29 mm | 4.74 (4.23 - 5.32) | <.001 |  | 3.18 (2.74 - 3.69) | <.001 |
| 30-39 mm | 6.33 (5.38 - 7.44) | <.001 |  | 4.24 (3.5 - 5.13) | <.001 |
| 40-49 mm | 4.75 (3.48 - 6.48) | <.001 |  | 2.97 (2.11 - 4.19) | <.001 |
| 50-59 mm | 6.53 (4.69 - 9.09) | <.001 |  | 4.32 (3.03 - 6.17) | <.001 |
| ≥ 60 mm | 6.49 (4.97 - 8.48) | <.001 |  | 3.76 (2.77 - 5.12) | <.001 |
| Pathological stage |  |  |  |  |  |
| I | – |  |  | – |  |
| II | 1.56 (1.36 - 1.77) | <.001 |  | 1.03 (0.89 - 1.21) | 0.676 |
| III | 2.31 (2 - 2.68) | <.001 |  | 0.8 (0.65 - 0.98) | 0.033 |
| IV | 5.28 (4.77 - 5.84) | <.001 |  | 1.53 (1.27 - 1.84) | <.001 |
| Radiotherapy |  |  |  |  |  |
| No | – |  |  | – |  |
| Yes | 2.73 (2.54 - 2.94) | <.001 |  | 0.76 (0.67 - 0.85) | <.001 |
| Alcohol consumption |  |  |  |  |  |
| No | – |  |  | – |  |
| Yes | 1.19 (1.11 - 1.28) | <.001 |  | 1.09 (1 - 1.18) | 0.042 |
| *Abbreviations: HR* Hazard ratio; *CI* Confidence interval. | | | | | |

Table S5. Results of Cox regression Model 3 (non-surgical model) for cancer-specific survival.

|  | Univariate | |  | Multivariate | |
| --- | --- | --- | --- | --- | --- |
|  | HR (95% CI) | *P* value |  | HR (95% CI) | *P* value |
| Age (per year) | 1.02 (1.02 - 1.02) | <.001 |  | 1.02 (1.02 - 1.02) | <.001 |
| Sex |  |  |  |  |  |
| Male | – |  |  | – |  |
| Female | 0.55 (0.49 - 0.62) | <.001 |  | 0.82 (0.71 - 0.94) | 0.005 |
| Body mass index (per score) | 0.91 (0.9 - 0.92) | <.001 |  | 0.94 (0.93 - 0.95) | <.001 |
| Diagnosis year (per year) | 0.72 (0.7 - 0.74) | <.001 |  | 0.72 (0.7 - 0.74) | <.001 |
| Tumor site |  |  |  |  |  |
| Oral cavity | – |  |  | – |  |
| Oropharynx | 0.42 (0.38 - 0.46) | <.001 |  | 0.58 (0.52 - 0.64) | <.001 |
| Hypopharynx | 0.49 (0.44 - 0.53) | <.001 |  | 0.64 (0.57 - 0.71) | <.001 |
| Nasopharynx | 0.15 (0.13 - 0.16) | <.001 |  | 0.32 (0.28 - 0.37) | <.001 |
| Larynx | 0.19 (0.15 - 0.22) | <.001 |  | 0.42 (0.34 - 0.51) | <.001 |
| Tumor size (per mm) | 1.02 (1.02 - 1.03) | <.001 |  | 1.01 (1.01 - 1.02) | <.001 |
| Maximum lymph node diameter |  |  |  |  |  |
| No metastasis | – |  |  | – |  |
| 1-9 mm | 0.98 (0.75 - 1.27) | 0.856 |  | 0.9 (0.68 - 1.18) | 0.447 |
| 10-19 mm | 1.28 (1.15 - 1.43) | <.001 |  | 0.86 (0.76 - 0.97) | 0.017 |
| 20-29 mm | 1.18 (1.06 - 1.32) | 0.003 |  | 1.00 (0.88 - 1.13) | 0.950 |
| 30-39 mm | 1.11 (0.98 - 1.26) | 0.100 |  | 1.06 (0.92 - 1.23) | 0.401 |
| 40-49 mm | 1.31 (1.12 - 1.53) | 0.001 |  | 1.27 (1.08 - 1.5) | 0.005 |
| 50-59 mm | 1.39 (1.19 - 1.62) | <.001 |  | 1.19 (1.01 - 1.4) | 0.041 |
| ≥ 60 mm | 2.00 (1.77 - 2.26) | <.001 |  | 1.59 (1.38 - 1.83) | <.001 |
| Clinical stage |  |  |  |  |  |
| I | – |  |  | – |  |
| II | 1.74 (1.28 - 2.38) | <.001 |  | 1.47 (1.06 - 2.05) | 0.020 |
| III | 2.42 (1.8 - 3.24) | <.001 |  | 2.15 (1.56 - 2.95) | <.001 |
| IV | 7.81 (5.92 - 10.31) | <.001 |  | 3.18 (2.34 - 4.32) | <.001 |
| Radiotherapy |  |  |  |  |  |
| No | – |  |  | – |  |
| Yes | 0.27 (0.25 - 0.3) | <.001 |  | 0.5 (0.45 - 0.55) | <.001 |
| Alcohol drinking |  |  |  |  |  |
| No | – |  |  | – |  |
| Yes | 1.59 (1.48 - 1.7) | <.001 |  | 1.16 (1.08 - 1.26) | <.001 |
| *Abbreviations: HR* Hazard ratio; *CI* Confidence interval. | | | | | |

Table S6. Results of calibration analysis for Model 2 (oral model) for overall mortality in different populations.

|  | Calibration Year | No. of cases | Observed | Predicted | Difference (%) | *P* value |
| --- | --- | --- | --- | --- | --- | --- |
| Training | 1 | 15,454 | 1,647 | 1,683 | 0.233 | 0.513 |
|  | 2 | 13,807 | 3,235 | 3,309 | 0.536 | 0.293 |
|  | 3 | 12,219 | 4,006 | 4,081 | 0.614 | 0.308 |
|  | 4 | 8,770 | 3,570 | 3,622 | 0.593 | 0.426 |
|  | 5 | 6,048 | 2,888 | 2,925 | 0.612 | 0.499 |
| Testing | 1 | 1,718 | 177 | 183 | 0.349 | 0.718 |
|  | 2 | 1,541 | 367 | 363 | -0.260 | 0.856 |
|  | 3 | 1,351 | 458 | 448 | -0.740 | 0.691 |
|  | 4 | 984 | 405 | 402 | -0.305 | 0.899 |
|  | 5 | 655 | 317 | 317 | 0.000 | 0.990 |
| Asian | 1 | 579 | 40 | 75 | 6.045 | <.001 |
|  | 2 | 547 | 66 | 118 | 9.506 | <.001 |
|  | 3 | 523 | 81 | 130 | 9.369 | <.001 |
|  | 4 | 494 | 87 | 134 | 9.514 | <.001 |
|  | 5 | 389 | 91 | 125 | 8.740 | 0.005 |
| White | 1 | 12,341 | 700 | 1,563 | 6.993 | <.001 |
|  | 2 | 11,424 | 1,315 | 2,421 | 9.681 | <.001 |
|  | 3 | 10,805 | 1,602 | 2,616 | 9.385 | <.001 |
|  | 4 | 10,105 | 1,761 | 2,681 | 9.104 | <.001 |
|  | 5 | 8,149 | 1,891 | 2,484 | 7.277 | <.001 |
| Black | 1 | 771 | 73 | 130 | 7.393 | <.001 |
|  | 2 | 714 | 122 | 197 | 10.504 | <.001 |
|  | 3 | 667 | 148 | 209 | 9.145 | <.001 |
|  | 4 | 634 | 163 | 217 | 8.517 | 0.001 |
|  | 5 | 525 | 168 | 203 | 6.667 | 0.023 |
| *Differences are calculated as ((predicted deaths - observed deaths) / number of cases) x 100. | | | | | | |

Table S7. Results of calibration analysis for Model 3 (non-surgical model) for overall mortality in different populations.

|  | Calibration Year | No. of cases | Observed | Predicted | Difference (%) | *P* value |
| --- | --- | --- | --- | --- | --- | --- |
| Training | 1 | 9,976 | 2,595 | 2,634 | 0.391 | 0.529 |
|  | 2 | 9,976 | 4,097 | 4,144 | 0.471 | 0.499 |
|  | 3 | 9,976 | 4,777 | 4,820 | 0.431 | 0.546 |
|  | 4 | 7,766 | 4,100 | 4,133 | 0.425 | 0.601 |
|  | 5 | 5,493 | 3,060 | 3,100 | 0.728 | 0.444 |
| Testing | 1 | 1,110 | 297 | 309 | 1.081 | 0.576 |
|  | 2 | 1,110 | 475 | 480 | 0.450 | 0.846 |
|  | 3 | 1,110 | 562 | 555 | -0.631 | 0.771 |
|  | 4 | 875 | 463 | 476 | 1.486 | 0.519 |
|  | 5 | 612 | 340 | 355 | 2.451 | 0.386 |
| Asian | 1 | 799 | 144 | 201 | 7.134 | <.001 |
|  | 2 | 796 | 224 | 310 | 10.804 | <.001 |
|  | 3 | 790 | 263 | 358 | 12.025 | <.001 |
|  | 4 | 768 | 285 | 377 | 11.979 | <.001 |
|  | 5 | 661 | 311 | 354 | 6.505 | 0.017 |
| White | 1 | 16,560 | 3,910 | 6,006 | 12.657 | <.001 |
|  | 2 | 16,514 | 5,781 | 9,068 | 19.904 | <.001 |
|  | 3 | 16,470 | 6,881 | 10,328 | 20.929 | <.001 |
|  | 4 | 16,244 | 7,633 | 10,943 | 20.377 | <.001 |
|  | 5 | 14,633 | 8,150 | 10,433 | 15.602 | <.001 |
| Black | 1 | 2,248 | 760 | 807 | 2.091 | 0.142 |
|  | 2 | 2,245 | 1,092 | 1,208 | 5.167 | 0.001 |
|  | 3 | 2,239 | 1,266 | 1,373 | 4.779 | 0.001 |
|  | 4 | 2,223 | 1,382 | 1,462 | 3.599 | 0.012 |
|  | 5 | 2,074 | 1,449 | 1,448 | -0.048 | 0.970 |
| *Differences are calculated as ((predicted deaths - observed deaths) / number of cases) x 100. | | | | | | |

Table S8. Results of calibration analysis for Model 1 (surgical model) for cancer-specific mortality in different populations.

|  | Calibration Year | No. of cases | Observed | Predicted | Difference (%) | *P* value |
| --- | --- | --- | --- | --- | --- | --- |
| Training | 1 | 20,315 | 1,334 | 1,289 | -0.222 | 0.364 |
|  | 2 | 19,050 | 2,366 | 2,221 | -0.761 | 0.022 |
|  | 3 | 18,284 | 2,767 | 2,541 | -1.236 | <.001 |
|  | 4 | 14,301 | 2,995 | 2,496 | -3.489 | <.001 |
|  | 5 | 10,922 | 3,108 | 2,276 | -7.618 | <.001 |
| Testing | 1 | 2,254 | 137 | 142 | 0.222 | 0.770 |
|  | 2 | 2,127 | 246 | 244 | -0.094 | 0.922 |
|  | 3 | 2,040 | 282 | 279 | -0.147 | 0.892 |
|  | 4 | 1,573 | 309 | 274 | -2.225 | 0.110 |
|  | 5 | 1,176 | 322 | 246 | -6.463 | <.001 |
| Asian | 1 | 1,036 | 62 | 120 | 5.598 | <.001 |
|  | 2 | 991 | 100 | 197 | 9.788 | <.001 |
|  | 3 | 951 | 133 | 220 | 9.148 | <.001 |
|  | 4 | 894 | 151 | 226 | 8.389 | <.001 |
|  | 5 | 712 | 158 | 212 | 7.584 | 0.001 |
| White | 1 | 24,752 | 1,315 | 3,272 | 7.906 | <.001 |
|  | 2 | 23,221 | 2,464 | 5,278 | 12.118 | <.001 |
|  | 3 | 22,099 | 3,035 | 5,829 | 12.643 | <.001 |
|  | 4 | 20,775 | 3,397 | 6,020 | 12.626 | <.001 |
|  | 5 | 16,828 | 3,661 | 5,611 | 11.588 | <.001 |
| Black | 1 | 2,028 | 172 | 300 | 6.312 | <.001 |
|  | 2 | 1,901 | 289 | 474 | 9.732 | <.001 |
|  | 3 | 1,800 | 365 | 521 | 8.667 | <.001 |
|  | 4 | 1,715 | 410 | 542 | 7.697 | <.001 |
|  | 5 | 1,427 | 435 | 512 | 5.396 | 0.002 |
| *Differences are calculated as ((predicted deaths - observed deaths) / number of cases) x 100. | | | | | | |

Table S9. Results of calibration analysis for Model 2 (oral model) for cancer-specific mortality in different populations.

|  | Calibration Year | No. of cases | Observed | Predicted | Difference (%) | *P* value |
| --- | --- | --- | --- | --- | --- | --- |
| Training | 1 | 14,774 | 967 | 940 | -0.183 | 0.515 |
|  | 2 | 13,887 | 1,668 | 1,574 | -0.677 | 0.079 |
|  | 3 | 13,368 | 1,920 | 1,772 | -1.107 | <.001 |
|  | 4 | 10,449 | 2,073 | 1,745 | -3.139 | <.001 |
|  | 5 | 7,967 | 2,152 | 1,599 | -6.941 | <.001 |
| Testing | 1 | 1,636 | 95 | 103 | 0.489 | 0.540 |
|  | 2 | 1,541 | 190 | 175 | -0.973 | 0.407 |
|  | 3 | 1,481 | 221 | 197 | -1.621 | 0.213 |
|  | 4 | 1,173 | 237 | 195 | -3.581 | 0.025 |
|  | 5 | 878 | 244 | 176 | -7.745 | <.001 |
| Asian | 1 | 579 | 40 | 76 | 6.218 | <.001 |
|  | 2 | 547 | 66 | 119 | 9.689 | <.001 |
|  | 3 | 523 | 81 | 131 | 9.560 | <.001 |
|  | 4 | 494 | 87 | 134 | 9.514 | <.001 |
|  | 5 | 389 | 91 | 126 | 8.997 | 0.005 |
| White | 1 | 12,341 | 700 | 1,564 | 7.001 | <.001 |
|  | 2 | 11,424 | 1,315 | 2,421 | 9.681 | <.001 |
|  | 3 | 10,805 | 1,602 | 2,616 | 9.385 | <.001 |
|  | 4 | 10,105 | 1,761 | 2,681 | 9.104 | <.001 |
|  | 5 | 8,149 | 1,891 | 2,485 | 7.289 | <.001 |
| Black | 1 | 771 | 73 | 130 | 7.393 | <.001 |
|  | 2 | 714 | 122 | 197 | 10.504 | <.001 |
|  | 3 | 667 | 148 | 210 | 9.295 | <.001 |
|  | 4 | 634 | 163 | 217 | 8.517 | 0.001 |
|  | 5 | 525 | 168 | 203 | 6.667 | 0.023 |
| *Differences are calculated as ((predicted deaths - observed deaths) / number of cases) x 100. | | | | | | |

Table S10. Results of calibration analysis for Model 3 (non-surgical model) for cancer-specific mortality in different populations.

|  | Calibration Year | No. of cases | Observed | Predicted | Difference (%) | *P* value |
| --- | --- | --- | --- | --- | --- | --- |
| Training | 1 | 8,852 | 1,471 | 1,350 | -1.367 | 0.012 |
|  | 2 | 8,024 | 2,145 | 1,894 | -3.128 | <.001 |
|  | 3 | 7,584 | 2,385 | 2,067 | -4.193 | <.001 |
|  | 4 | 6,212 | 2,529 | 2,058 | -7.582 | <.001 |
|  | 5 | 5,043 | 2,587 | 1,925 | -13.127 | <.001 |
| Testing | 1 | 969 | 156 | 153 | -0.310 | 0.865 |
|  | 2 | 875 | 240 | 214 | -2.971 | 0.153 |
|  | 3 | 812 | 264 | 230 | -4.187 | 0.064 |
|  | 4 | 689 | 277 | 233 | -6.386 | 0.014 |
|  | 5 | 557 | 282 | 216 | -11.849 | <.001 |
| Asian | 1 | 751 | 96 | 177 | 10.786 | <.001 |
|  | 2 | 725 | 153 | 247 | 12.966 | <.001 |
|  | 3 | 705 | 178 | 268 | 12.766 | <.001 |
|  | 4 | 677 | 194 | 277 | 12.260 | <.001 |
|  | 5 | 558 | 208 | 253 | 8.065 | 0.007 |
| White | 1 | 14,783 | 2,133 | 5,018 | 19.516 | <.001 |
|  | 2 | 13,834 | 3,101 | 6,643 | 25.604 | <.001 |
|  | 3 | 13,195 | 3,606 | 7,031 | 25.957 | <.001 |
|  | 4 | 12,489 | 3,878 | 7,110 | 25.879 | <.001 |
|  | 5 | 10,511 | 4,028 | 6,520 | 23.708 | <.001 |
| Black | 1 | 1,902 | 414 | 633 | 11.514 | <.001 |
|  | 2 | 1,729 | 576 | 808 | 13.418 | <.001 |
|  | 3 | 1,626 | 653 | 838 | 11.378 | <.001 |
|  | 4 | 1,548 | 707 | 850 | 9.238 | <.001 |
|  | 5 | 1,353 | 728 | 804 | 5.617 | 0.003 |
| *Differences are calculated as ((predicted deaths - observed deaths) / number of cases) x 100. | | | | | | |
